# Advanced Pharmaceutical Science and Technology in Israel

**DOI:** 10.3390/pharmaceutics17060734

**Published:** 2025-06-03

**Authors:** Arik Dahan

**Affiliations:** Department of Clinical Pharmacology, School of Pharmacy, Faculty of Health Sciences, Ben-Gurion University of the Negev, Beer-Sheva 8410501, Israel; arikd@bgu.ac.il; Tel.: +972-8-647-9483

## 1. Introduction

Israel has a significant and diverse history of ground-breaking research and development in pharmaceutical sciences and technology within academic settings, pharma/biopharma companies, and the biotechnology community [1].

This Special Issue of *Pharmaceutics*, entitled “Advanced Pharmaceutical Science and Technology in Israel”, was put together to highlight the exceptional achievements and significant international impact of pharmaceutical research performed in Israel in celebration of the country’s 75^th^ diamond anniversary of growth and prosperity.

In recent decades, the fields of pharmaceutical science and engineering have undergone rapid growth and development, both globally and in Israel alone [2]. To reflect this growth, we invited contributions on all aspects of pharmaceutical sciences and technology, including drug delivery, drug targeting, preformulation/formulation, biopharmaceutics/pharmacokinetics, nanopharmaceutics, pharmaceutics biotechnology, personalized medicine, and other related topics. We hoped that our Special Issue would contribute to multidisciplinary discussions on these exciting topics and prompt new scientific collaborations, further advancing pharmaceutical sciences and engineering in Israel and globally. In the next paragraphs, we summarize the contributions accepted for publication in Pharmaceutics under our Special Issue.

## 2. An Overview of Published Articles

In their article, Eydelman et al. developed a novel starch-based nanoparticle system that delivers cannabidiol (CBD) directly to the brain via intranasal administration. Compared to the CBD solution, the CBD-loaded nanoparticles achieved higher brain concentrations and showed strong anti-inflammatory effects in microglial cells, with low toxicity. These findings suggest that this system could be an effective method for targeting CBD treatment to the central nervous system (contribution 1).

The article by Bornstein et al. introduces melt-jet printing as a novel, solvent-free method of engineering active pharmaceutical ingredient (API) particles. Using paracetamol as a model, the researchers demonstrated how various substrates affect particle shape, with aluminum producing highly spherical microparticles due to its thermal and surface properties. The particles maintained their chemical integrity and exhibited minimal degradation. This method has also been successfully applied to other APIs, highlighting melt-jet printing’s potential for precise and reproducible pharmaceutical particle design (contribution 2).

The article by Ifrah et al. presents a novel oral drug delivery approach for the poorly absorbed antiviral drug zanamivir orally using self-double nanoemulsifying Winsor delivery systems (SDNE-WDS). Two formulations (SDNE-WDS1 and SDNE-WDS2) produced nanoemulsions that significantly enhanced zanamivir’s intestinal permeability, with particle sizes of 542.1 nm and 174.4 nm, respectively. Both systems increased drug transport across artificial membranes and in rat intestines, achieving permeability levels 3.5–5.5 times higher than the standard reference. These results highlight SDNE-WDSs as a promising strategy for improving the oral bioavailability of low-permeability, BCS class III drugs (contribution 3).

The article by Zinger et al. analyzed mechanical processing methods for extracting regenerative cells from lipoaspirate, aiming to identify the optimal technique without using enzymes. After reviewing 10,000 titles and selecting six relevant studies involving 117 patients, the researchers used multivariate meta-analysis to identify key factors affecting cell yield. Optimal conditions included a centrifuge force of 2000× *g* for 10 min, a cannula diameter of 2 mm, and 30 intra-syringe passes. Patient factors such as higher BMI and younger age were also associated with higher yield. The study highlights a novel statistical approach to optimizing mechanical lipoaspirate processing (contribution 4).

The article by Ramot et al. evaluated the Bioprotect biodegradable fillable balloon as a rectal spacer for prostate cancer radiotherapy using a rat model. Tissue responses at 4, 26, and 52 weeks post-implantation showed mild to moderate fibrosis and encapsulation, indicating good biocompatibility and safety. No adverse effects were observed, and all animals remained healthy. These results support the balloon’s potential for clinical use to reduce radiation-related complications and improve outcomes in prostate cancer treatment (contribution 5).

The article by Porat et al. investigated how bariatric surgery impacts the solubility, dissolution, and pharmacokinetics of sildenafil, the first-line drug to treat erectile dysfunction. Solubility tests showed a strong pH dependence, with significantly reduced solubility at the higher pH levels seen after gastric bypass surgery (especially post-OAGB). Dissolution and PBPK modeling revealed impaired absorption, delayed drug onset, and reduced peak concentrations (C_max_) in postbariatric patients, particularly after OAGB. These findings highlight the unpredictable and procedure-specific effects of bariatric surgery on sildenafil’s effectiveness, suggesting potential challenges in managing erectile dysfunction in this patient population (contribution 6).

The article by Ankri et al. evaluated the safety of Allocetra-OTS, a cell-based therapy using apoptotic cells, in a preclinical toxicology model with Sprague Dawley rats. Following three intravenous doses, no adverse effects were observed across key health indicators. Although temporary changes in immune cell and mild splenomegaly with extramedullary hematopoiesis occurred, these were expected and resolved during recovery. The highest tested dose was identified as the NOAEL. Overall, Allocetra-OTS was well tolerated, supporting its suitability for future clinical development (contribution 7).

The article by Merzbach et al. presents Clarstatin, a novel thiourea-bridged cyclic peptidomimetic designed to mimic a specific motif of the HLA-DRB1 gene’s shared epitope (SE). Clarstatin targets the proline-rich domain of cell surface calreticulin (CS-CRT), implicated in triggering autoimmune inflammation. Synthesized using Fmoc-SPPS and confirmed by LC-MS, Clarstatin demonstrated the ability to reduce calcium levels in Jurkat T cells and significantly alleviated symptoms in a mouse model of autoimmune uveitis. It was also found to be safe in acute toxicity tests, highlighting its potential as a therapeutic agent for treating inflammatory autoimmune diseases such as uveitis (contribution 8).

The article by Tepper-Shimshon et al. investigated how simulated microgravity (SMG) influences the cellular uptake of small molecules, reflecting microgravity-induced changes in membrane function. Using a random-positioning machine, researchers found that SMG increased uptake of efflux transporter substrates by up to 60% and reduced the uptake of a glucose transporter substrate by 20%. The uptake of a large cathepsin probe (MW 1198 g/mol) also increased, whereas the emission of molecules >3000 g/mol decreased by 50%. These results suggest that SMG can alter membrane transport differentially, potentially impacting drug distribution during spaceflight (contribution 9).

The article by Milošević et al. explored the use of a synthetic polymer (P-Esbp), presenting multiple E-selectin-binding peptides to inhibit leukocyte infiltration and reduce kidney inflammation. In acute kidney injury (AKI), P-Esbp effectively suppressed E-selectin expression and mitigated inflammation. However, in a chronic kidney disease (CKD) model, P-Esbp had a limited effect due to the compensatory actions of other adhesion molecules (P-selectin and VCAM-1). The findings highlight the complexity of cell adhesion mechanisms in kidney injury and suggest that targeting multiple CAMs could be a more effective strategy for controlling inflammation in both AKI and CKD (contribution 10).

## 3. Conclusions

Naturally, the articles published in this Special Issue represent only a small fraction of the top-notch pharmaceutical research and development taking place in Israel [3,4]. That being said, this collection of articles does serve as a unique example of the outstanding and diverse contributions Israel has made to the global pharmaceutical arena [5]. The Special Issue covers many hot topics in the pharmaceutical sciences, including gene therapy [6], cell-based therapy [7], nanomedicine [8], new drug delivery systems [9], 3D printing [10], space medicine [11], personalized medicine [12], and advanced PBPK modeling [13]. Looking to the future, Israel will undoubtedly continue to be a significant pioneer in the global pharmaceutical science and technology arenas.
